# Repurposing Artemisinin and its Derivatives as Anticancer Drugs: A Chance or Challenge?

**DOI:** 10.3389/fphar.2021.828856

**Published:** 2021-12-31

**Authors:** Zhaowu Ma, Clariis Yi-Ning Woon, Chen-Guang Liu, Jun-Ting Cheng, Mingliang You, Gautam Sethi, Andrea Li-Ann Wong, Paul Chi-Lui Ho, Daping Zhang, Peishi Ong, Lingzhi Wang, Boon-Cher Goh

**Affiliations:** ^1^ School of Basic Medicine, Health Science Center, Yangtze University, Jingzhou, China; ^2^ Department of Pharmacy, Faculty of Science, National University of Singapore, Singapore, Singapore; ^3^ Hangzhou Cancer Institute, Key Laboratory of Clinical Cancer Pharmacology and Toxicology Research of Zhejiang Province, Hangzhou, China; ^4^ Affiliated Hangzhou Cancer Hospital, Zhejiang University School of Medicine, Hangzhou, China; ^5^ Department of Pharmacology, Yong Loo Lin School of Medicine, National University of Singapore, Singapore, Singapore; ^6^ Cancer Science Institute of Singapore, National University of Singapore, Singapore, Singapore; ^7^ Department of Haematology–Oncology, National University Cancer Institute, Singapore, Singapore

**Keywords:** artemisinin, artemisinin derivatives, drug repurposing, anticancer therapy, pharmacokinetics, signalling pathways

## Abstract

Cancer has become a global health problem, accounting for one out of six deaths. Despite the recent advances in cancer therapy, there is still an ever-growing need for readily accessible new therapies. The process of drug discovery and development is arduous and takes many years, and while it is ongoing, the time for the current lead compounds to reach clinical trial phase is very long. Drug repurposing has recently gained significant attention as it expedites the process of discovering new entities for anticancer therapy. One such potential candidate is the antimalarial drug, artemisinin that has shown anticancer activities *in vitro* and *in vivo*. In this review, major molecular and cellular mechanisms underlying the anticancer effect of artemisinin and its derivatives are summarised. Furthermore, major mechanisms of action and some key signaling pathways of this group of compounds have been reviewed to explore potential targets that contribute to the proliferation and metastasis of tumor cells. Despite its established profile in malaria treatment, pharmacokinetic properties, anticancer potency, and current formulations that hinder the clinical translation of artemisinin as an anticancer agent, have been discussed. Finally, potential solutions or new strategies are identified to overcome the bottlenecks in repurposing artemisinin-type compounds as anticancer drugs.

## Introduction

Cancer has been a growing challenge in the healthcare system and is one of the largest global health problems. It is the second leading cause of death worldwide following ischemic heart disease. In 2018, the disease led to approximately 9.6 million deaths (Organisation 2018). An increase in cancer cases associated with aging population can increase the strain on the healthcare system and is certainly a cause for concern (Board, 2015).

Despite significant breakthrough in cancer therapy in the past decade, chemotherapy is still the mainstay of treatment (National Cancer Institute, 2015). Novel therapies such as targeted therapy and immunotherapy are not readily accessible owing to their high cost. In addition, targeted therapy often shows efficacy in specific cancers exhibiting selected biomarkers in a small group of patients and the majority of cancer patients do not respond to immunotherapy (Ventola, 2017). Therefore, there is still an unmet demand to develop more effective and cheaper anticancer drugs and identify the lead compounds for the development of those drugs.

The cost to develop a novel cancer drug is extremely high and the process from target identification to phase III clinical trials is time-consuming. Therefore, drug repurposing is becoming an increasingly explored alternative approach to the traditional drug discovery and development pipeline (Lim et al., 2021; Ren et al., 2021). Since data on existing drugs are largely available, additional studies on its pharmacology, pharmacokinetics and safety are not required (Sleire et al., 2017). Thus, drug repurposing can greatly reduce the duration of the drug development process and time to reach the market as an oncology therapeutic (Parvathaneni et al., 2019), greatly reducing the cost and increasing the patients’ access to the treatment (Parvathaneni et al., 2019).

**FIGURE 1:**
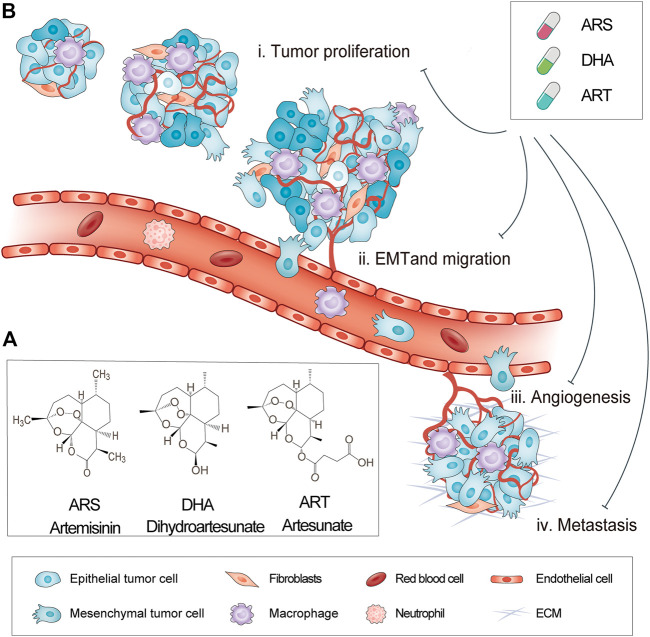
Chemical structure and anticancer activity of artemisinins. **(A)** Chemical structure of artemisinins **(B)** Multifunctional anticancer activity of artemisinins.

One group of compounds that is currently being explored for drug repurposing is artemisinin (ARS) and its derivatives (henceforth referred to as artemisinins). Artemisinins are sesquiterpene trioxanes (Figure 1A) that have been clinically used to treat malaria (Augustin et al., 2020; Wang et al., 2020; Wang et al., 2021). The maximum recommended dose is 200 mg daily for 3 days for oral therapy of uncomplicated malaria (Organization, 2015). This dosing regimen has been shown to be safe and effective for the treatment of malaria. However, cancer is a chronic condition that may require long-term treatment with artemisinins in contrast to an acute infection like malaria. In addition, cancer treatment may require a higher dose of the drug-to be effective, leading to higher levels of toxicity than that observed in malaria treatment. For the treatment of severe malaria 2.4 mg/kg IV artesunate (ART) administered at 0, 12, and 24 h for up to 7 days is recommended, which is a considerably higher than that required to treat uncomplicated malaria, and adverse reactions of delayed hemolysis at this dose have been reported (Prevention, 2020, May 28). It is unclear whether such side effects will be more prominent at doses used for cancer treatment because no dosing regimen has yet been established for cancer treatment. Therefore, the safety of artemisinins in long-term cancer therapy requires further investigation.

Artemisinins have shown potent anticancer activity in multiple cancers (Wong et al., 2017) (Figure 1B). Artemisinins, ART, and dihydroartemisinin (DHA) exhibited therapeutic effects against multiple tumor types such as breast cancer (Zhang et al., 2015; Yao Y. et al., 2018; Wen et al., 2018), prostate cancer (Xu et al., 2016; Zhou et al., 2017), ovarian cancer (Wu et al., 2012; Zhou et al., 2020), pancreatic cancer (Zhou et al., 2013), and lung cancer (Zhou et al., 2012; Zuo et al., 2014). Artemisinins acts against cancer cells *via* various pathways such as inducing apoptosis (Zhu et al., 2014; Zuo et al., 2014) and ferroptosis *via* the generation of reactive oxygen species (ROS) (Zhu et al., 2021) and causing cell cycle arrest (Willoughby Sr et al., 2009; Tin et al., 2012). Therefore, artemisinins can work on multiple targets and affect multiple signaling pathways (Wong et al., 2017). Moreover, ARS has been known to be well tolerated and safe at low doses, lowering the risk of intolerable toxicity (Efferth, 2017). Thus, artemisinins show great potential of repurposing as anticancer drugs.

While most studies showed *in vitro* and *in vivo* anticancer efficacy of artemisinins, limited clinical trials in human subjects have been conducted to date. Therefore, the practicality of clinical translation of artemisinins as anticancer agents is uncertain. This review outlines the potential anticancer activity of artemisinins. Additionally, the pharmacokinetic properties of artemisinins, one of the most important aspects in anticancer drug development are discussed in details. This review article will improve our understanding of the limitations in the development of artemisinins as anticancer drugs in human subjects and suggest potential solutions and new strategies to overcome those challenges.

## Search Strategy

**FIGURE 2:**
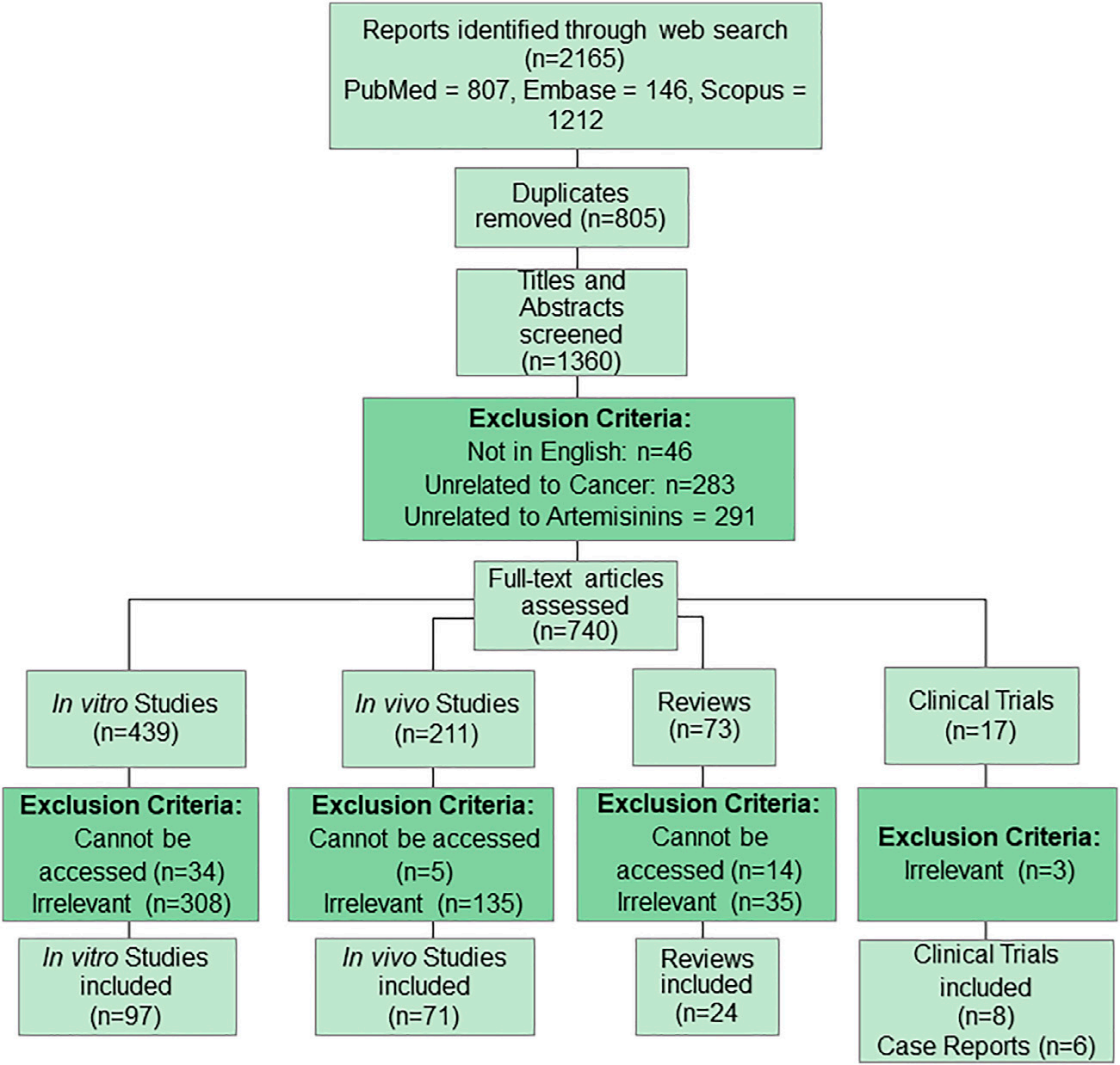
Primary search strategy for anticancer properties of artemisinins.

We performed a literature search on PubMed, Scopus, and embase. The first search aimed to identify studies on anticancer effect of artemisinins; thus the search terms (“artemisinins” [Mesh] AND “Neoplasms” [Mesh]) OR ((artemisinin [Title/Abstract]) AND (cancer [Title/Abstract]) were used. The search strategy is illustrated in Figure 2.

Another search was performed to understand the pharmacokinetic properties of artemisinins and the following search terms were used ((“artemisinins” [Mesh]) OR ((artemisinin [Title/Abstract]) AND ((“Pharmacokinetics” [Mesh]) OR (pharmacokinetic [Title/Abstract])). Duplicates were removed using Endnote and titles and abstracts were screened according to the exclusion criteria as illustrated in Figure 2.

## Pharmacokinetics of Artemisinins

It is important to understand a drug’s pharmacokinetic properties to determine its potential for clinical use. Many studies have been conducted to determine the pharmacokinetic parameters of artemisinins. The main pharmacokinetic characteristics of artemisinins namely absorption, distribution, metabolism, and excretion are elaborated in *Absorption of Artemisinins*–*Elimination of Artemisinins*.

### Absorption of Artemisinins

An AUC_0-∞_ value (area under the curve from time 0 extrapolated to infinite time) of 657 μg h L^−1^ was observed in a study on healthy volunteers administered orally 4 mg/kg of ART (Na-Bangchang et al., 2004). To calculate absolute bioavailability, this value was compared to that of another study on healthy volunteers administered 4 mg/kg IV dose of ART (AUC_0-∞_ value of 3,038 μg h L^−1^) (Li et al., 2009). Therefore, absolute bioavailability was estimated to be 21.6%. In contrast, the AUC_0-∞_ value of a group of patients with uncomplicated malaria who received 200 mg oral ART was considerably high (4,868 μg h L^−1^), indicating that disease condition may affect absorption (Newton et al., 2002) because patients with malaria experience greater exposure than that of healthy volunteers, as indicated by the AUC_0-∞_ values.

To better understand the translational potential of artemisinins as anticancer agents, maximun concentration (C_max_) values also evaluated. C_max_ values of DHA ranged between 0.558–1.270 μM in healthy volunteers (Teja-isavadharm et al., 2001; Na-Bangchang et al., 2004). In healthy volunteers who received oral ART, C_max_ values ranged between 0.174–1.830 μM (Teja-isavadharm et al., 2001; Batty et al., 2002; Na-Bangchang et al., 2004; Diem Thuy et al., 2008; Li et al., 2009). Moreover, C_max_ values were compared with IC_50_ values of promising cancer cell lines obtained *in vitro* to understand the limitaions in clinical translation. Compared to healthy volunteers, patients with uncomplicated malaria showed high C_max_ values of 3.9–4.6 μM for the use of ART (Binh et al., 2001; Newton et al., 2002) and 3.7–4.03 μM for DHA (Binh et al., 2001; Newton et al., 2002). Thus, the disease state affects the absorption of artemisinins, and further studies are required to better understand the pharmacokinetics of artemisinins in cancer patients.

### Distribution of Artemisinins

Artesunate has been reported to have small volume of distribution (Vd/F) of 0.0106–0.0920 L/kg because ART has good solubility and is not lipophilic [28]. Therefore, ART would not distribute well to the tissues and might be more effective in treating cancers such as leukemia, hepatocellular carcinoma (HCC), or renal cell carcinoma because the liver and kidney are highly perfused organs. Artesunate might also be useful for the treatment of metastatic cancers. A low Vd/F also implies a short elimination half-life (t_1/2_). In contrast, ARS was recorded to have a much higher Vd/F ranging from 33.7 ± 16.1 to 38.4 ± 18.9 L/kg (Ashton et al., 1998) because ARS is more lipophilic and less water soluble than ART. However, ARS is converted to the active metabolite DHA in the body, which has good solubility with Vd/F of 1.46 L/kg reported in metastatic breast cancer patients (Ericsson et al., 2014).

### Elimination of Artemisinins

Pharmacokinetic studies showed a relatively short t_1/2_ of artemisinins. For ART, t_1/2_ was 0.41 h (Teja-isavadharm et al., 2001) after an oral dose of 100 mg in healthy volunteers. At a dose of 4 mg/kg, t_1/2_ of 0.74 h was reported (Na-Bangchang et al., 2004). Generally, t_1/2_ has been reported to be less than 1 h and dose-dependent; however, the variations in t_1/2_ with dose are not drastic. A low t_1/2_ value aligns with a low Vd/F value, which implies that a more frequent dosage regimen is required for anticancer treatment with ART because it is cleared from the body relatively quickly. The oral clearance of ART was reported to be 20.6 ± 10.6 L/h/kg (Teja-isavadharm et al., 2001) for 100 mg oral dose, which is considerably high. Because of its high solubility, ART is eliminated by the kidneys. It is important to understand the metabolism and clearance of a drug to determine the recommended dose. However, to successfully determine a dosage regimen, the desired C_max_ value should be identified.

The challenges in repurposing artemisinins as anticancer drugs can be overcome by using different formulations and combination therapies based on pharmacokinetic properties of these drugs.

## Mechanisms of Action Underlying Anticancer Activity of Artemisinins

**FIGURE 3:**
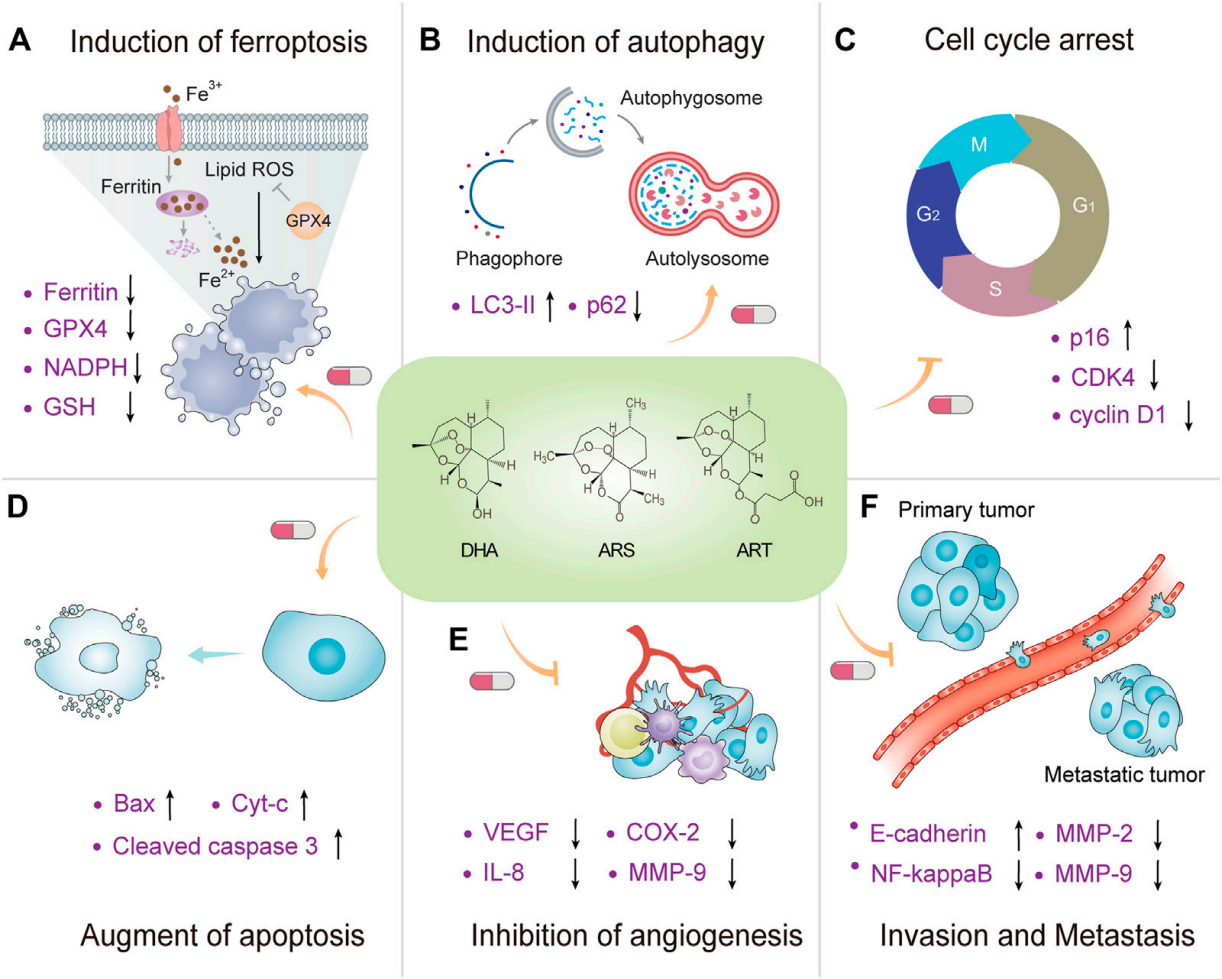
Mechanisms of action underlying anticancer activity of artemisinins. A schematic view of the molecular crosstalk pathways involved anticancer mechanisms of artemisinins, including **(A)** induction of ferroptosis, **(B)** induction of autophagy, **(C)** cell cycle arrest, **(D)** augment of apoptosis, **(E)** inhibition of angiogenesis, and **(F)** invasion and metastasis.

Artemisinins possess anti-cancer activity, although the underlying mechanisms remain unclear. Generally, artemisinins act *via* similar pathways because they have a special structure called peroxide bridge, which is strongly associated with the cytotoxicity required for their antimalarial and anticancer activities (Liao et al., 2014; Tran et al., 2014; Xu et al., 2015; Tong et al., 2016). A cell death model revealed a distinguished anticancer mechanism of artemisinins through induction of ferroptotic cell death (Zhu et al., 2021). Other common mechanisms of action include induction of autophagy, cell cycle arrest, and apoptosis. Inhibition of cell proliferation and metastasis was observed in both *in vitro* and *in vivo* studies (Hou et al., 2008; Michaelis et al., 2010; Wang et al., 2012; Tran et al., 2014; Xu et al., 2015; Tong et al., 2016) (Figure 3). Hence, multiple signalling pathways are involved in anticancer activities of artemisinins in various cancer types. This section focuses on common mechanisms, which are further detailed in Table 1.

**TABLE 1:** IC_50_ and Mechanisms of artemisinins *in vitro*.

Cancer type	Cell line	IC_50_ value (µM)			Mechanism of action	Ref
		24H	48H	72H		
**Artemisinin**						
Gall bladder Cancer	GBC-SD	—	49.1 ± 1.69	—	Upregulate p16, downregulate CDK4 and cyclin D1 to induce G1-phase cell cycle arrest	Jia et al. (2016a)
	NOZ	—	58.6 ± 1.77	—		
					Activate caspase-3 to induce apoptosis	
					Induce Δψm collapse of *via* cytochrome c release	
					Induce the generation of ROS inhibition of cell motility and migration	
HCC	HepG2	—	10.4	250	Dose- and time-dependent	Weifeng et al. (2011)
	SMMC-7721	—	—	290		
	HepG2	—	14.0	—		Hou et al. (2008)
					Inhibit invasion and metastasis of HCC cells	
	BEL7407		9.90			
	Huh-7		8.90		Suppress p-p38, ERK1/2 activation in HCC cells	
					Inhibit cell invasion by altering MMP2 and TIMP2 balance	
					Activate Cdc42 to increase adhesion and decrease metastasis	
					Induce G1-phase cell cycle arrest	
					Increase production of Cip1/p21 and Kip1/p27	
					Downregulate CDKs and cyclins	
					Induce apoptosis by inducing change in the expression of apoptosis related proteins	
Lung Cancer	A549	—	—	—	Regulate metastasis, migration, and invasion by suppressing EMT and CSCs	Tong et al. (2016)
					Depress Wnt/β-catenin signaling pathway	
					Inhibit cyclin D1 to induce G1-phase cell cycle arrest and suppress cell viability	
	H1299	—	—	—		
	NCI-H292	—	—	—	Induce deprivation of cysteine and inhibit GPX4 to increase sensitivity of the cancer cells to ferroptosis in a time- and dose- dependent manner	
Breast Cancer	MDA-MB-453	—	—	—		(Yao et al., 2018b; Chen et al., 2020a)
	MCF7	—	—	—		
Colon Cancer	HCT116	—	>80.0	—		
					Induce production of ROS by reacting with iron	
	SW480	—	>80.0	—		
	HT29	—	>80.0	—		
Endometrial Cancer	Ishikawa	—	—	—	Inhibit CDK-4 and induce G1-phase cell cycle arrest	Tran et al. (2014)
					Disrupt NF-κB binding to the artemisinin responsive region of the CDK4 promoter	
					Disrupt NF-κB subunit p65 and p50 localization into the cell nuclei	
					Promote interaction between p65-IκB-α and p50-IκB-α	
Rhabdomyosarcoma	TE671	—	—	—	Generation of ROS	Beccafico et al. (2015)
	RD18	—	—	—		
**Dihydroartemisinin**						
Myeloid Leukaemia	K562	—	11.3	—	Induce autophagy	Wang et al. (2012)
					Upregulate ROS levels intracellularly	
					Induce apoptosis by activating caspase cascade	
Pancreatic Cancer	BxPC-3	—	—	40.6 ± 6.8	Induce G0/G1 cell cycle arrest in a dose-dependent manner	(Chen et al., 2010; Wang et al., 2011)
	AsPC-1	—	—	—		
					Decrease NF-κB/p65 expression	
	PANC-1	—	—	48.9 ± 6.1	Inhibit NF-κB and downregulate VEGF, IL-8, COX-2, and MMP-9	
					Reduce DNA-binding activity of NF-κB/p65 and promote antiangiogenic activity	
Hepatocellular Carcinoma	HepG2	—	13.4	—	Induce G1-phase cell cycle arrest	Hou et al. (2008)
	Hep3B	—	10.3	—	Increase production of Cip1/p21 and Kip1/p27	
	Huh-7	—	9.6	—		
	BEL-7404	—	9.3	—	Downregulate CDKs and cyclins	
					Induce apoptosis by inducing change in the expression of apoptosis related proteins	
Lung Cancer	A549	—	—	—	Induce apoptosis	(Liao et al., 2014; Tong et al., 2016)
	H1229	—	—	—	Block cell cycle progression from G1 to S phase by suppressing cyclin D1 expression	
					Regulate metastasis, migration, and invasion by suppressing EMT and CSCs	
					Depress Wnt/β-catenin signaling pathway	
					Suppress cell viability	
Ovarian Cancer	OVCA-420	—	5.64 ± 0.33	—	Inhibit cell growth in a dose- and time-dependent manner	(Jiao et al., 2007; Chen et al., 2009b)
	OVCA-439	—	3.83 ± 0.14	—	Induce apoptosis by targeting the Bcl-2 family	
	OVCA-433	—	4.48 ± 0.21	—	Decrease expression of Bcl-2 and Bcl-xL which are antiapoptotic proteins	
	OVCAR-10	—	5.72 ± 0.07	—		
					Increase Bax and Bad promoter proteins	
					increase PARP	
					Activate caspases	
	HEY	—	5.51 ± 0.27	—		
					Induce G2-phase cell cycle arrest	
	OVCA-432	—	14.0 ± 0.50	—		
	OVCAR-3	—	14.9 ± 0.28	—		
	OCC-1	—	13.8 ± 0.53	—		
	SK-OV-3	—	14.6 ± 0.42	—		
	ALST	—	15.2 ± 0.37	—		
Fibrosarcoma	HT-1080 cells	—	—	—	Inhibit MMP-9 and MMP-2 transcription and expression, hence suppressing PMA-induced invasion and migration	Hwang et al. (2010)
					Suppress PMA-stimulated NF-κB and AP-1	
					Work through PKC, ERK, and JNK signalling pathway to suppress PMA-mediated invasion	
					Block PKCα/Raf/MAPKs and NF-κB/AP-1 signaling pathways	
Head and Neck Squamous Cell Carcinoma	Fadu	85.4	25.7	—	Inhibit constitutive phosphorylation and activation of STAT3	Jia et al. (2016b)
	HEp-2	41.4	24.5	—		
					Selectively block phosphorylation of Jak2	
	Cal-27	44.7	9.70	—		
Rhabdomyosarcoma	TE671	50.0	—	—	Generation of ROS	Beccafico et al. (2015)
					Induce apoptosis	
	RD18	—	—	—		
Neuroblastoma	UKF-NB-3	4.50 ± 0.30	—	—	Induce apoptosis by activating caspase-3	Michaelis et al. (2010)
	UKF-NB-6	6.24 ± 0.19	—	—		
Lung cancer	NCI-H292	—	—	—	Increase degradation of ferritin by lysosomes causing an increase in free iron in cells leading to sensitisation to ferroptosis	(Yao et al., 2018b; Chen et al., 2020a)
Colon Cancer	HCT116	—	1.20	—		
	HT29	—	1.25	—		
					Regulate iron homeostasis *via* signalling between iron regulatory protein (IRP) and iron-responsive element (IRE)	
	SW480	—	1.25	—		
	LOVO	—	1.20	—		
	RKO	—	1.80	—		
					Inhibit GPX4 and cause cysteine deprivation	
Breast Cancer	MDA-MB-453	—	—	—	Increase sensitivity of cells to RSL3-induced cell death	
**Artesunate**						
Cervical Cancer	HeLa	5.47	25.7	-	Induce cytotoxicity	Luo et al. (2014)
					Increase radiosensitivity of HeLa, but not SiHa	
					Induce apoptosis and necrosis in HeLa	
	SiHa	6.34	24.5	—		
Breast Cancer	MCF-7	—	—	—	Upregulate expression of Beclin1	(Hamacher-Brady et al., 2011; Chen et al., 2014b; Chen et al., 2020a)
	MDA-MB-231	—	—	—	Induce autophagy	
					Suppress cell viability through autophagy	
	T47D	—	—	—		
	MDA-MB-453	—	—	—		
					Induce G2/M-phase cell cycle arrest Cause lysosomal mitochondrial	
					fragmentation	
					Activate cell death of MCF-7	
Neuroblastoma	UKF-NB-3	2.69 ± 0.10	—	—	Activate caspase-3 to induce apoptosis	Michaelis et al. (2010)
					Induce oxidative stress	
	UKF-NB-6	3.54 ± 0.42	—	—		
Kaposi’s Sarcoma	KS-IMM	—	—	—	Induce apoptosis	Dell'Eva et al. (2004)
					Suppress angiogenesis	
Ovarian Cancer	HEY1	—	—	5.80 ± 1.62	Induce ROS	Greenshields et al. (2017)
					Inhibit cell division and induce cell cycle arrest	
	HEY2	—	—	7.34 ± 0.56		
	IGROV-1	—	—	8.82 ± 1.18	Modulate cell cycle regulatory protein expression and mTOR signalling	
	OVCAR8	—	—	5.51 ± 1.06		
					ROS and iron-dependent cytotoxicity	
					Cause ROS-dependent G2/M-phase	
	OVCAR3	—	—	15.0 ± 6.38		
					cell cycle arrest	
	SKOV-3	—	—	23.6 ± 3.86		
					Cause ROS-independent G1-phase cell cycle arrest	
	TOV-21G	—	—	6.11 ± 0.64		
					Interfere with mTORC1 signalling by inhibiting phosphorylation of downstream p70 S6K1 and S6 ribosomal protein	
	OV-90	—	—	31.9 ± 4.15		
	TOV-112D	—	—	0.51 ± 0.03		
					Work through caspase-dependent and caspase-independent pathways	
	HO8910	—	—	—	Induce ROS and DNA double-strand	
	A2780	—	—	—	Downregulate RAD51 to increase sensitivity to cisplatin	
	HEY		—	—		
					Sensitise cells to cisplatin by acting synergistically with cisplatin to induce double-stranded breaks	
					Inhibit formation of RAD51 foci induced by cisplatin	
Pancreatic Cancer	MiaPaCa-2	—	—	—	Induce caspase-independent and non-apoptic cell death	(Youns et al., 2009; Du et al., 2010)
	BxPC-3	—	279.3	—		
					Induce change in mitochondrial membrane potential and ROS-mediated cell death	
	Panc-1	—	26.8	—		
	CFPAC-1	—	142.8	—		
					Inhibit growth and proliferation	
					Induce apoptosis	
					Induce activation of caspase 3 and caspase 7	
					Potentiate effect of gemcitabine in growth inhibition	
Renal Cell Carcinoma	Caki-1	—	—	6.70	Induce G2/M-phase cell cycle arrest	Jeong et al. (2015)
	786-O	—	—	11.0	Induce cell death by generation of ROS and depletion of intracellular depletion of ATP	
	SN12C-GFP	—	—	23.0		
Rhabdomyosarcoma	TE671	10.0	—	—	Induce apoptosis by causing ROS production	Beccafico et al. (2015)
	RD18	10.0	—	—		
					Induce expression of myo-miRs, miR-133a and miR-206 that is reliant on ROS and independent of p38	
Osteosarcoma	HOS		52.8		Inhibit proliferation	Xu et al. (2011)
					Induce G2/M phase cell cycle arrest	
Leukaemia	J-Jhan	—	—	1.33 ± 0.14	Induce G2/M-phase cell cycle arrest	Steinbrück et al. (2010)
	J16	—	—	4.39 ± 0.44	Induce apoptosis via generation of ROS	(Efferth et al., 2007; Steinbrück et al., 2010)
	SKM-1	61.2	38.4	28.6	Inhibit proliferation	Xu et al. (2015)
					Induce apoptosis	
					Enhance cell adhesion and inhibit metastasis *via* the Wnt/β-catenin pathway by blocking translocation of subcelluar β-catenin and E-cadherin to adherent junctions of the membrane	
					Enhance chemosensitivity to other agents	
	CEM	∼0.10	-	—	Generate ROS and induce apoptosis *via* the intrinsic pathway	Efferth et al. (2007)
	Molt-4	—	∼0.50	—		
					Synergise with doxorubicin to	
	Hut78	—	∼6.0	—	enhance apoptosis	
	Parental Jurkat A3	—	∼2.0	—		
Lung Cancer	H69	—	—	2.54 ± 0.23	Induce G2/M-phase cell cycle arrest	Steinbrück et al. (2010)
	H1299	—	—	—	Inhibit migration, invasion, and metastasis by suppressing EMT and CSCs	Tong et al. (2016)
	A549	—	100	—		
					Suppress Wnt/β-catenin pathway	
					Inhibit cyclin D1 to induce G1-phase cell cycle arrest	
					Suppress cell viability	

	H1395	—	150	—	Inhibit proliferation	Rasheed et al. (2010)
	LXF289	—	60.0	—		
					Inhibit u-PA activity, protein and mRNA expression	
	H460	—	7.50	—	Inhibit transactivating capacity of NF-κB	
	Calu3	—	10.0	—		
					Inhibit AP-1 transcription factors	
	H1299	—	12.5	—	Regulate transcription of MMP-2, MMP-7 and u-PA.	
					Regulate invasion and metastasis	
	NCI-H292	—	—	—	Increase sensitisation to ferroptosis	Chen et al. (2020a)
Colon Cancer	HCT116	2.20	—	29.9 ± 2.49	Inhibit cell viability	(Steinbrück et al., 2010; Chen et al., 2017b; Chen et al., 2020a)
					Inhibit biosynthetic of fatty acid	
					Induce apoptosis via mitochondrial pathway activation and lipid ROS production	
					Inhibit NF-κB pathway	
					Induce G2/M-phase cell cycle arrest	
					Inhibit u-PA activity, protein and	
	CLY	—	—	20.3 ± 2.20	Inhibit proliferation most strongly in CLY, followed by Lovo, then HT-29	(Li et al., 2008; Chen et al., 2020a)
	Lovo	—	—	30.6 ± 0.73		
					Promote apoptosis	
					Induce G2/M-phase cell cycle arrest most prominently in HT-29	
	HT-29	—	—	82.3 ± 3.74		
					Induce S-phase cell cycle arrest most prominently in CLY.	
	SW480	—	—	—		
					Inhibit hyperactive Wnt pathway	
					Increase sensitisation to ferroptosis	
Hepatocellular Carcinoma	HepG2	—	20.5	—	Huh-7 and Hep3B: induce ROS-dependent apoptosis	(Hou et al., 2008; Zeng and Zhang, 2011; Pang et al., 2016)
	Hep3B	—	39.4	—		
					HepG2: induce ROS-independent apoptosis	
	BEL7404	—	15.0	—		
					Reduce cell viability	
	Huh-7	—	9.22	—	Alkylate haem-harbouring nitric oxide synthase in a dose-dependent manner to mitigate proliferation	
Glioblastoma	U251	—	—	73.3 ± 1.32	Induce apoptosis and necrosis	(Steinbrück et al., 2010; Berdelle et al., 2011)
					Induce oxidative DNA damage	
	LN-229	—	—	—	Induce G2/M-phase cell cycle arrest	
Melanoma	SK-Mel-28	—	—	94.4 ± 2.93	Induce apoptosis	Steinbrück et al. (2010)
Prostate Cancer	DU145	—	—	70.5 ± 5.81 µM	Induce apoptosis	Steinbrück et al. (2010)

### Induction of Ferroptosis

Ferroptosis, an oxidative, iron-dependent form of regulated cell death, is characterized by the accumulation of ROS and lipid peroxidation products to lethal levels (Stockwell et al., 2017). Emerging evidence suggests that triggering ferroptosis is a promising therapeutic strategy to kill cancer cells, particularly for eradicating aggressive malignancies that are resistant to the traditional therapies (Liang et al., 2019). Compared to normal cells, ferritin, a major iron storage protein essential for iron homeostasis, is overexpressed in many cancer cells (Buranrat and Connor, 2015). Usually, high ferritin level in blood is a poor prognostic marker in cancer patients, leading to aggressive disease. Other endogenous molecules such as glutathione, nicotinamide adenine dinucleotide phosphate, and glutathione peroxidase 4 (GPX4) have been also closely linked to the regulation of ferroptosis (Stockwell et al., 2017).

Dihydroartemisinin renders cancer cells more sensitive to ferroptosis by increasing the cellular accumulation of free ions due to its ability to induce lysosomal degradation of ferritin in an autophagy-independent manner (Chen X. et al., 2020). Dihydroartemisinin augmented GPX4 inhibition-induced ferroptosis in some cancer cells in both *in vitro* and *in vivo* models by the inducible knockout of GPX4 (Chen X. et al., 2020). Du *et al.* revealed that DHA, the main active metabolite of ART, could be a promising therapeutic agent to preferentially target acute myeloid leukemia cells by inducing ferroptosis (Du et al., 2019). Jiang *et al.* demonstrated that ART could regulate the labile iron pool (LIP) by promoting the lysosomal degradation of ferritin through lysosomal acidification, thereby inducing ROS-dependent cell death in HCC cells. The accumulation of labile iron in the endoplasmic reticulum promoted excessive ROS production and severe endoplasmic reticulum disruption, leading to cell death. These findings suggest ART is a safe anti-HCC agent that disturbs iron homeostasis (Jiang et al., 2021). Besides, artesunate greatly enhanced the anticancer effects of low dose of sorafenib against HCC by inducing oxidative stress and lysosome-mediated ferritinophagy, two essential aspects of ferroptosis (Li ZJ. et al., 2021). Furthermore, Hamacher-Brady *et al.* demonstrated that ART could trigger programmed cell death (PCD) in cancer cells in a manner dependent on the level of free iron and the generation of ROS (Hamacher-Brady et al., 2011). Moreover, artesunate could inhibit autophagosome turnover and cause perinuclear clustering of autophagosomes, early and late endosomes, and lysosomes. Lysosomal iron was the lethal source of ROS upstream of mitochondrial outer membrane permeabilization because lysosomal iron chelation blocked all measured parameters of ART-induced PCD, whereas lysosomal iron loading enhanced death. Two lysosomal inhibitors, chloroquine and bafilomycin A1, reduced ART-induced PCD, proving that lysosomal function is required in the process of PCD signaling (Hamacher-Brady et al., 2011). The anticancer effect of ART can be attributed, at least partially, to ferroptosis.

### Induction of Autophagy

Emerging evidence suggests that autophagy induction is one of the molecular mechanisms underlying anticancer activity of artemisinins (Wang et al., 2012; Chen K. et al., 2014). Mitochondria are important molecular organelles that regulate both apoptosis and autophagy (type II PCD), and ROS generation is one of the triggering factors for mitochondrial dysfunction. DHA-induced autophagy in leukemia K562 cells, evidenced by LC3-II protein expression, was observed to be ROS-dependent (Wang et al., 2012). Inhibitory effect of DHA on the proliferation of leukemia K562 cells was also dependent upon the iron level, indicating an association between autophagy and ferroptosis (Wang et al., 2012).

In a study on breast cancer cells, ART could inhibit the proliferation of cancer cells by inducing autophagy [53]. Moreover, ART sensitized breast cancer cells to epirubicin chemotherapy. As a result, ART was regarded as a therapeutic candidate in breast cancer therapy [53]. A recent study evaluated the antineoplastic effects of ART in diffuse large B cell lymphoma cells (Chen et al., 2021). The results revealed that ART exhibited anticancer activity through multiple mechanisms of action including autophagy as evidenced by over-expression of LC3B-I/II, whereas p62 expression was downregulated in a dose dependent manner following 24 h of ART treatment. Next, Chen, *et al.* investigate the antitumor activity of DHA in esophagus cancer cells (Chen X. et al., 2020). The results showed that DHA could inhibit the migration capacity of Eca109 and TE-1 cells by inducing autophagy. Ma *et al.* also demonstrated similar results that DHA significantly reduced the viability of Eca109 cells in a dose- and time-dependent manner (Ma et al., 2020) Together, these studies indicate that autophagy is one of the key mechanisms underlying death of cancer cells treated with artemisinin and its derivatives.

### Induction of Cell Cycle Arrest

Artemisinins administration resulted in cell cycle arrest in a dose-dependent manner (Willoughby Sr et al., 2009; Chen et al., 2010; Wang et al., 2011). G_1_-phase cell cycle arrest was observed in GBC-SD and NOZ gallbladder cancer cell lines (Jia J. et al., 2016), LNCaP, PC3, and DU145 prostate cancer cells (Steinbrück et al., 2010), A549 and H1299 lung cancer cells (Liao et al., 2014; Tong et al., 2016), BxPC-3 and AsPC-1 pancreatic cancer cells (Chen et al., 2010), human hepatoma cells (Hou et al., 2008), ovarian cancer cells (Greenshields et al., 2017) and human Ishikawa endometrial cancer cells (Tran et al., 2014).

The induction of G_1_-phase cell cycle arrest by artemisinins is mediated by several pathways, including downregulation of cyclin-dependent kinase 4 (CDK4) and cyclin D1 expression (Hou et al., 2008; Liao et al., 2014; Tran et al., 2014; Jia J. et al., 2016; Tong et al., 2016), both of which promote cell proliferation. Moreover, artemisinin enhanced the expression of p16 (Jia J. et al., 2016), a tumor suppressor that inhibits CDK and limits cell cycle progression. G_2_/_M_-phase cell cycle arrest was also observed in other cell lines including J-Jhan, HCT116, H69, U251 (Steinbrück et al., 2010), human osteosarcoma (Xu et al., 2011), breast cancer (Chen K. et al., 2014), and renal carcinoma (Jeong et al., 2015) cells following the administration of ART. In renal carcinoma cells and ovarian cancer cells, ART-mediated G_2_/_M_-phase cell cycle arrest was dependent on ROS generation (Jeong et al., 2015; Greenshields et al., 2017). In breast cancer cells, ART caused G_2_/_M_-phase cell cycle arrest by regulating autophagy (Chen K. et al., 2014). Cell cycle arrest is one of the key molecular mechanisms of anticancer activity of artemisinins.

### Augmentation of Apoptosis

Artemisinins have been reported to induce apoptosis in J16, DU145, SK-Mel-28 (Steinbrück et al., 2010), leukaemia (Efferth et al., 2007; Zhou et al., 2007), HepG2, Hep3B hepatoma (Hou et al., 2008), ovarian cancer (Jiao et al., 2007), Kaposi’s sarcoma-IMM (Dell'Eva et al., 2004), cervical cancer (Luo et al., 2014), SKM-1 (Xu et al., 2015), glioblastoma (Berdelle et al., 2011), neuroblastoma (Michaelis et al., 2010), embryonal rhabdomyosarcoma (Beccafico et al., 2015), pancreatic cancer (Youns et al., 2009), and colorectal cancer (Li et al., 2008; Chen X. et al., 2017) cells. Similar to the cell cycle arrest, apoptosis induction was caused by a myriad of signaling pathways.

One common pathway by which artemisinins induced apoptosis is the generation of ROS which in turn damages organelles, DNA, and proteins, eventually leading to the death of cancer cells (Efferth et al., 2007; Beccafico et al., 2015; Pang et al., 2016; Chen X. et al., 2017). ROS-dependent apoptosis caused by Bax-mediated intrinsic pathway has been observed in Huh-7 and Hep3B cells following treatment with ART (Pang et al., 2016), in which caused mitochondrial activation, and release of cytochrome c and subsequent activation of caspase-9. leading to activation of caspase-3, an executioner caspase that destroys cellular structures such as poly (ADP-ribose) polymerase, an enzyme involved in DNA repair, causing cell death (Hou et al., 2008; Jia J. et al., 2016; Chen X. et al., 2017). In another study, exposure to artemisinins led to a dose dependent increase in caspase-3 cleavage in HepG2 cells (Hou et al., 2008). This process was also evident in K562 leukemia (Zhou et al., 2007) and pancreatic cancer cells (Youns et al., 2009). However, activation of caspase-3 is not always ROS-dependent. Both *in vitro* and *in vivo* studies have also shown that ART could induce ROS-independent apoptosis in HepG2 cells (Pang et al., 2016).

### Inhibition of Angiogenesis

Angiogenesis is a key factor in tumor growth, invasion and metastasis. It is partly mediated by the transcription factor NF-κB and pro-angiogenic factors (including VEGF, IL-8, COX-2 and MMP-9) (Ferrara and Kerbel, 2005; Liu et al., 2021). Dihydroartemisinin showed anti-angiogenic effect in both *in vitro* angiogenesis models and *in vivo* pancreatic cancer-derived tumor models (Wang et al., 2011). These effects were likely to be mediated by inhibiting the NF-κB pathway and its downstream pro-angiogenic growth factors. In this study, the results showed that treatment of human umbilical vein endothelial cells with DHA resulted in a dose-dependent inhibition of cell proliferation and capillary tube formation.

The pleiotropic transcription factor NF-κB regulates the expression of multiple genes, including VEGF and IL-8 (Huang et al., 2000). The constitutive NF-κB activity drives the constitutive overexpression of VEGF and IL-8, which contributes to the angiogenic phenotype of human pancreatic cancer. After DHA treatment, decreased expression of VEGF and IL-8 *in vitro* and *in vivo* is associated with decreased proliferation and neovascularization.

Artesunate can inhibit the expression of VEGF, which is closely related to the level of VEGF secreted in the conditioned medium. Artesunate has potential anti-leukemia effects for the treatment for cronic myeloid leukemia or as an adjunct to standard chemotherapy regimens (Zhou et al., 2007). Using KS-IMM cells derived from Kaposi’s sarcoma lesions of kidney transplant patients, Dell E’va *et al.* proved that ART could inhibit the growth of cancer cells and normal human umbilical cord endothelial cells (Dell'Eva et al., 2004) ART also reduces angiogenesis *in vivo* in terms of vascularization of Matrigel plugs injected subcutaneously into syngeneic mice. In summary, ART is a promising low-cost drug candidate for the treatment of hyper vascularized Kaposi’s sarcoma. and for preventing tumor angiogenesis.

### Inhibition of the Key Signaling Pathways

NF-κB is a transcription factor that regulates apoptosis, and promotes tumorigenesis, cell proliferation, metastasis, and angiogenesis upon activation (Chen H. et al., 2009). Hence, inhibition of the NF-κB pathway may block these processes and result in cell apoptosis. In BxPC-3 and PANC-1 pancreatic cancer cells, DHA inhibited NF-κB and decreased the production of vascular endothelial growth factor (VEGF), IL-8, COX-2, and MMP-9 (Wang et al., 2011), promoting angiogenesis. NF-κB activates cyclin D1 and Bcl-2 transcription. DHA inhibited both Bcl-2 and cyclin D1 (Chen H. et al., 2009), which are the downstream gene products of NF-κB. The disruption of the NF- B pathway at different points was also observed in HCT116 (Chen X. et al., 2017) and lung cancer cells (Rasheed et al., 2010), after ART administration, HT-1080 cells (Hwang et al., 2010) after DHA administration, and human Ishikawa endometrial cancer cells (Tran et al., 2014) after ARS administration.

Tong *et al.* demonstrated that ARS, DHA and ART induced cell cycle arrest in the G1 phase, thereby inhibiting the proliferation of A549 and H1299 cells. Moreover, artemisinins inhibited other malignant tumor markers by migration, invasion, cancer stem cells and epthelial-mesenchymal transition (EMT) and decreased tumor growth in xenograft mouse model. Using IWP-2, Wnt/β-catenin pathway inhibitor and Wnt5a siRNA, Tong *et al.* showed that anticancer effect of artemisinins partly depends on the inactivation of the Wnt/β-catenin signaling. Artemisinin significantly reduced the protein levels of Wnt5-a/b, and increased the levels of NKD2 and Axin2, and ultimately inhibited the Wnt/β-catenin pathway (Tong et al., 2016). Xu *et al.* demonstrated that ART induced SKM-1 cell apoptosis in a dose- and time-dependent manner by inhibiting the hyperactive β-catenin signaling pathway (Xu et al., 2015).

Artemisinins inhibit cell proliferation and metastasis (Hou et al., 2008; Xu et al., 2015; Tong et al., 2016). Inhibition of the Wnt/β-catenin pathway in lung cancer by DHA and SKM-1 cells by ART led to increased E-cadherin expression (Xu et al., 2015; Tong et al., 2016), which mediates cell-cell adhesion. The increased cell-cell adhesion suppressed tumor metastasis (Xu et al., 2015). In a human fibrosarcoma HT-1080 cell model, anti-invasive effect of DHA was caused by inhibiting the phosphorylation of PKCalpha/Raf/ERK and JNK and reducing the activation of NF-κB and AP-1, thereby leading to the down-regulation of MMP-9 expression. Therefore, DHA is an effective anti-metastatic agent that works by down-regulating MMP-9 expression (Hwang et al., 2010). In another study on HepG2 cells, ARS activated Cdc42, promoting E-cadherin action which is necessary for cell adhesion (Weifeng et al., 2011). Additionally, artemisinins administration downregulated proliferating cell nuclear antigen gene expression, MMP2, p-p38, p-ERK1/2, CSC markers, and EMT-related proteins, which promote tumor growth, proliferation, and metastasis in lung cancer and HCC cells and their downregulation would inhibit tumor growth (Rasheed et al., 2010; Weifeng et al., 2011; Liao et al., 2014; Tong et al., 2016). Artemisinins inhibited proliferation in prostate cancer, human osteosarcoma, HepG2, and pancreatic cancer cells (Willoughby Sr et al., 2009; Youns et al., 2009; Xu et al., 2011; Zeng and Zhang, 2011).

Overall, artemisinins act *via* multipe pathways by regulating the key targets of suppression of cell cycle, induction of apoptosis, inhibition of NF-κB signalling pathway, and suppression of mitogen-activated protein kinase (MAPK) signaling.

## Anticancer Efficacy of Artemisinins *in Vitro* and *in Vivo* Models

Artemisinins have been recognized as antimalarials, but they have demonstrated great anticancer potential in *in vitro* and *in vivo* studies (Table 2).

**TABLE 2:** Dose and Mechanisms of Action of artemisinins *in vivo*.

Animal	Dosing regimen	Disease model	Mechanisms, safety, and efficacy	Reference
**Drugs: artemisinin**				
Male BALB/c nude mice	100 mg/kg per day orally over 30 days	GBC-SD and NOZ-derived gallbladder cancer xenograft mouse models	Inhibitory effect on GBC cell-derived tumours	Jia et al. (2016b)
			Reduce tumour volume and weight	
			Inhibit cell proliferation	
Male BALB/c athymic nude mice	100 mg/kg per day orally	LNCaP prostate cancer xenograft model	Inhibit proliferation of LNCaP cells *in vivo*	Willoughby Sr et al. (2009)
			Inhibited growth of LNCaP xenografts	
			Reduce tumour size and volume	
			Tumours showed no gross vascularity and looked pale yellow, like avascular tissue	
			No adverse side effects observed	
Nude BALB/c mice	C0: 0 mg/kg/day C1: 50 mg/kg/day C2: 100 mg/kg/day with stepwise increase in dose	HepG2 hepatocellular carcinoma orthotopic xenograft	Inhibit metastasis	Weifeng et al. (2011)
			Reduce number of tumours found in lungs as compared to the control group	
			Tumour inhibition rate:	
			C1: 51.8%	
			C1: 51.8%	
Female BALB/c-nude mice	60 mg/kg/day	A549 NSCLC xenograft model	Inhibition of tumour growth	Tong et al. (2016)
			Reduce tumour weight and volume	
			Did not cause significant weight loss	
Female athymic nude mice	50 mg/kg/day OR 100 mg/kg/day OR combination with gemcitabine	HepG2 hepatocellular carcinoma xenograft model	Inhibit tumour growth (30.0 and 39.4% for 50 mg/kg/d and 100 mg/kg/d)	Hou et al. (2008)
			increase anticancer effect of gemcitabine	
			No observable toxic effects	
Female athymic nude mice	50 mg/kg/day OR 100 mg/kg/day OR combination with gemcitabine	Hep3B hepatocellular carcinoma xenograft model	Inhibit tumour growth slightly	Hou et al. (2008)
			Combination with gemcitabine does not increase inhibition of tumour growth	
			Induce G1-phase arrest and apoptosis	
**Drugs: Dihydroartemisinin**				
Female Balb/c-nude mice	60 mg/kg/day	A549 NSCLC xenograft model	Decrease tumour volume and weight significantly	Tong et al. (2016)
			No significant body weight loss	
Male nude BALB/c mice	2 mg/kg/day 10 mg/kg/day 50 mg/kg/day i.p. injection for 21 days	BxPC-3 pancreatic cancer xenograft	Slow tumour growth	Wang et al. (2011)
			Decrease tumour volume	
			2 mg/kg/day: 569 ± 69 mm^3^	
			5 mg/kg/day: 389 ± 44 mm^3^	
			10 mg/kg/day: 244 ± 36 mm^3^	
			Control: 730 ± 90 mm^3^	
			Decrease microvessel density significantly	
			Inhibit angiogenesis	
Female athymic nude mice	50 mg/kg/day OR 100 mg/kg/day OR combination with gemcitabine	HepG2 hepatocellular carcinoma xenograft model	Inhibit tumour growth (36.1 and 60.6% for 50 mg/kg/d and 100 mg/kg/d)	Hou et al. (2008)
			Increase anticancer effect of gemcitabine	
			No observable toxic effects	
Female athymic nude mice	50 mg/kg/day OR 100 mg/kg/day OR combination with gemcitabine	Hep3B hepatocellular carcinoma xenograft model	Inhibit tumour growth	Hou et al. (2008)
			Increase antitumour effect when combined with gemcitabine	
			Induce G1-phase cell cycle arrest	
			Induce apoptosis	
Male nude BALB/c mice	10 mg/kg/day i.p. injection OR combination with gemcitabine 100 mg/kg BD	BxPC-3 pancreatic cancer xenograft model	Reduce tumour volume and suppress tumour growth	Wang et al. (2010a)
			Combination treatment reduced tumour volume more significantly	
			Decrease Ki-67	
			Suppress NF-κB DNA binding activity and downregulate related gene products	
			Enhance antitumour effect of gemcitabine	
BALB/c male mice	50 mg/kg/day, 5 times per week, for 4 weeks	Cal-27 head and neck squamous cell carcinoma xenograft	Decrease tumour size, volume, and weight significantly	Jia et al. (2016b)
			No significant body weight loss	
Female athymic nude Foxn1nu/Foxn1+ mice	5 mg/kg/day OR in combination with DOX diet intraperitoneal injection	GPX4 iKO H292 lung cancer xenograft model	Suppress tumour growth	Chen et al. (2020a)
			Decrease expression of Ki-67	
			Enhance effect of GPX4 targeted therapy	
**Drugs: Artesunate**				
Female Balb/c-nude mice	60 mg/kg/day	A549 NSCLC xenograft model	Inhibit tumour growth to decrease tumour volume and weight significantly	Tong et al. (2016)
			Did not cause significant loss in body weight	
Female BALB/c-nu mice	50 mg/kg/day 100 mg/kg/day 200 mg/kg/day i.p. injection 18 days	HOS human osteosarcoma xenograft model	Inhibit tumour growth dose-dependently and reduce tumour volume	Xu et al. (2011)
			Caused some decrease in body weight	
Female BALB/c athymic nude mice	25 mg/kg/day 50 mg/kg/day 100 mg/kg/day	Panc-1 pancreatic cancer xenograft model	Suppress tumour growth	Du et al. (2010)
			25 mg/kg/day: 33%	
			50 mg/kg/day: 44%	
			100 mg/kg/day: 65%	
			Well tolerated and no observable toxicity	
Female C57BL/6 mice	100 mg/kg i.p. injection	ID8 murine ovarian cancer model	Inhibit tumour growth and reduce tumour size	Greenshields et al. (2017)
			No overt toxicity or significant loss in body weight	
C57BL/6 &Male (CD-1) BR nude mice	167 mg/kg/day	KS-IMM xenograft model	Suppress tumour growth and reduce tumour weight significantly	Dell'Eva et al. (2004)
Male outbred BALB/c mice	100 mg/day OR in combination with radiation therapy	HeLa and SiHa cervical cancer xenograft	Inhibit growth of HeLa xenografts in combination with irradiation	Luo et al. (2014)
			Enhance radiosensitivity of HeLa xenograft	
			Did not significantly change radiosensitivity of SiHa xenograft	
Athymic BALB/c male nude mice	50 mg/kg/day oral	HN9 head and neck cancer xenograft model	Inhibit tumour growth	Roh et al. (2017)
			Synergise with trigonelline to suppress tumour growth	
			Decrease GSH and increase γH2AX	
Female BALB/c nude mice	100 mg/kg/day i.p. injection	786-O renal cell carcinoma xenograft model	Exert antitumour effect and inhibit tumour growth	Jeong et al. (2015)
			Prevent angiogenesis and metastasis	
			decrease Ki-67 to curb proliferation	
Female athymic nude mice	50 mg/kg alone OR in combination with cisplatin 2 mg/kg for 16 days	A2780 and HO8910 ovarian cancer xenografts	Synergise with cisplatin to inhibit tumour growth	Wang et al. (2015)
			ARS alone did not exhibit significant antitumour effect	
Female athymic nu/nu mice	25 mg/kg/day i.p. injection	TE671 embryonal rhabdomyosarcoma xenograft model	Significantly inhibit tumour growth (50% reduction in mass)	Beccafico et al. (2015)
			Reduce % of cells in mitotic phase (H3r + ve cells)	
			Increase expression of pho-p38 and decrease levels of myogenin and PAX7	
			Did not affect body weight	
-	Artesunate i.v. injected for metastasis essay or applied on upper CAM	Chicken embryo metastasis (CAM) model	Inhibit metastasis (decreased number of metastasised cells)	Rasheed et al. (2010)
			Suppress tumour growth and reduce tumour size on upper CAM.	
			Downregulate MMP-2, MMP-7, and u-PA mRNA.	
			Inhibit invasion	
Female athymic nude mice	300 mg/kg twice a week	HT29, CLY, and Lovo colorectal cancer xenografts	Suppress tumour growth	Li et al. (2008)
			CLY tumour growth inhibitory rate = 50.5%	
			Lovo tumour growth inhibitory rate = 52.2%	
			HT29: less significant inhibition, HT29 less sensitive to artesunate	
Athymic nu/nu female mice	50 mg/kg OR 100 mg/kg OR 200 mg/kg i.p. 3 times a week for 4 weeks	KBM-5 chronic myeloid leukaemia xenograft model	Suppress tumour growth	Kim et al. (2015)
			Downregulate Ki-67 expression	
			Downregulate VEGF expression	
			Activate caspase-3	
			Inhibit p38, ERK, CREB, STAT5, and JAK2 phosphorylation	
			Suppress apoptosis proteins expression such as bcl-2, bcl-xL, IAP-1/2	
			Induce expression of proteins bax and p21	

### *In vitro* Anticancer Efficacy

Several studies have been conducted to assess the effect-of artemisinins against different types of cancer. For DHA, IC_50_ values ranged between 1.20–15.2 μM (Jiao et al., 2007; Hou et al., 2008; Chen T. et al., 2009; Michaelis et al., 2010; Wang et al., 2012), with the exception of BxPC-3 pancreatic cancer cells (Chen et al., 2010; Wang et al., 2011), TE671 rhabdomyosarcoma cells (Beccafico et al., 2015) and Fadu, Hep-2, and Cal-27 head and neck squamous cancer cells (Jia L. et al., 2016) which were highly resistant. This IC_50_ range was considerably higher than that of C_max_ in healthy volunteers (0.558–1.27 μM). Only HCT116, HT29, SW480, and LOVO colon cancer cell lines showed IC_50_ values within the C_max_ range (Yao Z. et al., 2018; Chen GQ. et al., 2020). For ART, IC_50_ values range bewteen 2.0–39.4 μM (Efferth et al., 2007; Hou et al., 2008; Li et al., 2008; Youns et al., 2009; Du et al., 2010; Michaelis et al., 2010; Rasheed et al., 2010; Steinbrück et al., 2010; Zeng and Zhang, 2011; Luo et al., 2014; Beccafico et al., 2015; Jeong et al., 2015; Xu et al., 2015; Pang et al., 2016; Greenshields et al., 2017; Chen GQ. et al., 2020). Inconsistent with the range of C_max_ values (0.174–1.83 μM) except in CEM, J-Jhan and Molt-4 leukemia cells (Efferth et al., 2007; Steinbrück et al., 2010), and TOV-112D ovarian cancer cells (Greenshields et al., 2017) which are within range. High IC_50_ value is a significant barrier in the clinical application of use of artemisinins in humans because high doses *in vivo* may lead to toxicity problems. Combination therapy can also be considered as a therapeutic option because artemisinins can synergize with other drugs to increase efficacy.

### *In Vivo* Anticancer Efficacy

Several studies demonstrated the efficacy of artemisinins in tumor-bearing animal models. The cancer types identified *in vitro* have been effectively treated by artemisinins *in vivo*. The *in vivo* studies used more aggressive dosage regimens of artemisinins with effective doses ranging from 50 to 100 mg/kg/dand showed little toxicity in animals (Hou et al., 2008; Willoughby Sr et al., 2009; Du et al., 2010; Wang et al., 2011; Weifeng et al., 2011; Xu et al., 2011; Jeong et al., 2015; Jia L. et al., 2016; Jia et al., 2016b; Tong et al., 2016). In HepG2 HCC xenografts, tumor inhibition rates of up to 79.6% was observed after administration of 100 mg/kg/d of ARS (Weifeng et al., 2011). Another study repoted 60.6% inhibition of tumor growth after administration of 100 mg/kg/d of DHA (Hou et al., 2008). Since HCC cell lines were not highlighted in previous *in vitro* studies, the underlying mechanism of the efficacy of DHA observed in HCC xenografts *in vivo* should be further explored.

At this dosage range, artemisinins showed a significant and conclusive effect on the inhibition of tumor growth. However, 100 mg/kg/d dose would translate to 3 g/d for a 60 kg adult, which is significantly greater than the safe and effective dose established for the treatment of malaria (200 mg/d) (Organization, 2015). Another promising result was observed in LOVO colorectal cancer xenografts where the tumor growth inhibition rate was 52.2% (Li et al., 2008) at a dose of 300 mg/kg twice a week. This discrepancy in dosage regimens between malaria cases and *in vivo* studies in xenograft mouse models can make clinical translation challenging.

Notably, among many derivatives of artemisinin, ART has the most extensive data, thus, it has the greatest potential to be developed for future use in cancer treatment in humans.

## Clinical Application of Artemisinins in Cancer Therapy

A few clinical trials conducted were using ART to understand the efficacy of artemisinins in breast cancer, colorectal cancer, and other solid tumors (Table 3) (Krishna et al., 2015; von Hagens et al., 2017; Deeken et al., 2018). The effective dose of ART ranged up to 200 mg/d, which was safe and well tolerated (von Hagens et al., 2017; von Hagens et al., 2019).

**TABLE 3:** Human clinical trials of artemisinins.

Study design and population	Dosing regimen	Efficacy data	Safety data	Ref
Phase 1 open label study 23 patients with metastatic breast cancer	Oral ART 100 mg OD OR 150 mg OD OR 200 mg OD Add on to guideline-based oncological therapy4 weeks	No complete or partial remission	Oral ART 200 mg/d (2.2–3.9 mg/kg/d) was well tolerated and safe	ARTIC M33/2 (von Hagens et al., 2017)
		10 patients were found to have stable disease (considered as a clinical benefit)		
			72 AEs that were possibly related to ART were recorded	
		5 patients experienced progression		
			86.1% of AEs possibly related to ART were resolved at the time of last study visit	
Prospective monocentric, and open uncontrolled phase I dose-finding study 13 patients with metastatic breast cancer for long-term compassionate use	Oral ART 100 mg OD OR 150 mg OD OR 200 mg OD Add-on therapy to guideline-based oncological therapy	6 patients 150 or 200 mg OD (1.8–3.3 mg/kg BW/d), were found to have stable disease until last follow-up	No major safety concerns	von Hagens et al. (2019)
			6 patients experienced grade 3 adverse events possibly related to ART.	
		4 patients taking 100 mg OD (<2 mg/kg/d) experienced progression		
		2 patients taking 150 mg OD (2.1–2.7/kg/d) experienced progression		
		1 patient taking 200 mg OD (3.9–4.1 mg/kg/d) experienced progression		
		Longest treatment period reached with 150 mg OD (1.8–2.7 mg/kg/d)		
Randomised, Double Blind, Placebo-Controlled Pilot Study 23 patients with colorectal cancer 12 received treatment, 11 received placebo	Oral ART 200 mg/d for 14 days	Decreased expression of Ki-67 (probability = 97%)	6 patients had adverse events, 2 were possibly related to ART.	Krishna et al. (2015)
		Increased expression of CD31 (probability = 79%)		
		Increased recurrence-free survival probability compared to placebo after 3 years (0.89 vs 0.5)		
		No patients that received ART had increased carcinoembryonic antigen (CEA) levels as compared to the placebo group where 3 patients had increased CEA levels		
			2 patients who were at the lower weight limit of inclusion developed leukopenia	
Phase I 19 adult patients with refractory solid tumours	IV ART 8, 12, 18, 25, 34 and 45 mg/kg given on days 1 and 8 of a 21-days cycle administered as a 5-min IV push	No patients had complete or partial response	18 mg/kg on a Day1/Day8, 3-weeks administration cycle was shown to be the maximum tolerated dose	Deeken et al. (2018)
			C_max_ at the maximum tolerated dose was 415 ng/ml	
			Dose limiting toxicities included myelosuppression, liver dysfunction, uncontrolled nausea and vomiting, hypersensitivity	
			Side effects of anaemia, fatigue, N&V, anorexia, dizziness reported	
		4 patients had stable disease, 3 of which had ampullary, renal, and ovarian cancers. They were on the 18, 12, and 8 mg/kg dose levels respectively		
		The other with stable disease was on the 18 mg/kg dose and experienced a 10% reduction in tumour measures		
Dose-escalation phase I study 28 women with cervical intraepithelial neoplasia 2/3 (CIN2/3)	Intravaginal ART Group 1: one treatment cycle of 50 mg inserts. Next 3 groups: 1, 2, or 3 treatment cycles of 200 mg insert(s), at weeks 0, 2, and 4 of the study Each treatment cycle included a single vaginal insert dose for 5 nights in a row	Histologic regression to CIN1 or less observed in 68% of subjects	No intolerable side effects that led to withdrawal	Trimble et al. (2020)
			No grade 3 or 4 adverse events reported	
		>60% histologic regression across all 4 dosing groups		
			3 participants reported no noticeable side effects	
		Mean time to regression shorter in subjects that received multiple treatment cycles compared to only one		
			Treatment generally safe and well-tolerated	
Phase I 120 patients with advanced NSCLC	Control: vinorelbine + cisplatin (NP) Treatment: NP + artesunate 120 mg/day	No significant difference in short-term survival rate, mean survival time	Toxicity between treatment and control group not significantly different	Zhang et al. (2008)
		disease controlled rate significantly higher in treatment group		
		Time to progression significantly longer in treatment group		
2 patients with metastatic uveal melanoma in addition to standard chemotherapy	Artesunate on compassionate use basis	One patient experienced temporary response upon adding ART to Fotemustine	Well tolerated with no experience of additional side effects	Berger et al. (2005)
		The other patient experienced stabilistation and regression of spleen and lung metastases		
		Promising adjuvant in treatment of melanoma		

A clinical trial conductedin patients with solid tumors revealed the maximum tolerated dose of IV ART as 18 mg/kg in a Day 1/Day 8 regimen with a 3-week administration cycle with dose-limiting toxicities such as myelosuppression, liver dysfunction, and uncontrolled nausea and vomiting (Deeken et al., 2018). Other side effects included anemia, fatigue, dizziness, and anorexia (Deeken et al., 2018) at a much lower dose than the effective dose used in *in vivo* studies. This result indicates that *in vivo* studies do not accurately represent toxicity data in humans. While effective therapeutic range *in vivo* can be as high as 200 mg/kg/d, the same dose cannot be used in humans. Caution should be exercised in proceeding with higher doses of ART that are likely to be more efficacious but less safe.

Another study showed anticancer activity of ART in colorectal cancer patients, which is consistent with the previous *in vitro* and *in vivo* studies (Li et al., 2008; Chen GQ. et al., 2020). Treatment with 200 mg oral ART increased recurrence-free survival rate compared to placebo after 3 years (Krishna et al., 2015), but two patients at the lower weight limit developed leukopenia.

The ARCTIC M33/2 study conducted in patients with metastatic breast cancer used ART as an adjuvant to the patients’ guideline-based cancer therapy for 4 weeks; 10 out of 23 patients had stable disease, whereas five patients experienced disease progression (von Hagens et al., 2017). Therefore, while 200 mg oral ART has been established as a relatively safe dose, efficacy at this dose remains inconclusive. The ARCTIC M33/2 study was extended for long-term compassionate use in 13 patients who did not experience any clinically relevant adverse events in the original phase I study. Results from the follow-up study suggested the dose dependent effects of ART; a greater number of patients adminitered lower dose (100 mg/kg/d) experienced disease progression than patients administered higher doses (von Hagens et al., 2019). In some patients, up to 37 months of use of ART has been reported, demonstrating the safety of the long-term use of oral ART at this dosage range.

Few clinical trials that have been conducted to date are limited to phase I trials which involved relatively small study populations. Hence, phase II trials are required to investigate the effect of artemisinins on a larger number of patientsand gain better insight into the safety and efficacy of the use of artemisinins, in particular ART, as potential anticancer agents in large populations.

## Future Perspectives

Artemisinins, in particular ART, have been proven to promising drugs to repurpose for cancer treatment. Additional phase II and III trials should be conducted in future to gain a better understanding of the long-term safety and efficacy profile of artemisinins in large populations. Further strategies should be explored to expedite the development of artemisinins as anticancer agents.

### Combination Therapy

Combination therapy makes use of multiple agents to treat a single condition, a strategy that is commonly employed in cancer treatment. The use of combination therapy has advantages of synergistic and additive effects because different drugs can work on different molecular pathways to exert a greater anticancer effect, thereby leading to greater efficacy. Since IC_50_ values of artemisinins cancer treatment are relatively high, combination therapy can be used to take advantage of the synergistic effect and lower IC_50_, and minimise any dose-related toxicities because combination therapy allows the use of lower doses of multiple agents.

Several drugs have demonstrated synergistic effects *in vitro* when administered in combination with either DHA or ART or both (Table 4). Many studies also reported that the use of artemisinins sensitized cancer cells to conventional chemotherapy and exerted a synergistic effect on apoptosis, inhibition of cell growth, and a reduction of cell viability, leading to a lower IC_50_ value (Chen T. et al., 2009; Zhang YJ. et al., 2013; Liu and Cui, 2013; Shen et al., 2016; Tai et al., 2016; Chen H. et al., 2017; Wang et al., 2017; Yang et al., 2019a; Yang et al., 2019b; Hu et al., 2019). Combination index, which measures the degree of drug interactions (Zhang JL. et al., 2013) was used to understand the potential of combination therapy. The combination of DHA with cisplatin (Zhang YJ. et al., 2013), DHA with onconase (Shen et al., 2016), DHA with gemcitabine (Yang et al., 2019a), DHA with Apo2L/TRAIL (Kong et al., 2012), and ART with sorafenib (Yao et al., 2020), which were used to treat lung, lung, ovarian, pancreatic, and liver cancer, had combination index values < 1, which indicates synergism.

**TABLE 4:** Promising combination therapies of artemisinins.

Agent combined with DHA/ART	Cell line/disease model	Effect	Ref
**Drugs: Dihydroartemisinin**			
Onconase	MSTO-211H human mesothelioma	Significant synergistic antitumour effects with onconase	Shen et al. (2016)
		Drastic decrease in IC_50_ values from onconase or DHA monotherapy to combination therapy. In SK-MES-1 cells, IC_50_ value of both dropped from ∼1,200 to ∼10 µM. In Spc-A-1 cells, IC_50_ value of onconase was as low as 0.001 µM when administered together with DHA.	
	NCI-H661, SK-MES-1, SPC-A-1, and A549 NSCLC cells		
Doxorubicin	Hep3b hepatocellular carcinoma cells	increase apoptosis-inducing effects of doxorubicin	Yang et al. (2019b)
		Inhibit P-gp expression which causes resistance to doxorubicin	
	MCF-7 breast cancer cells	Combination therapy activated caspase cascades more than monotherapy	Wu et al. (2013)
		DHA sensitised apoptosis triggered by doxorubicin	
	HeLa cervical cancer, OVCAR-3 ovarian, MCF-7 breast, PC-3 prostate, and A549 lung cancer cells	Decrease cell viability	Tai et al. (2016)
		Synergistic effect to induce apoptosis	
Gemcitabine	A2780 ovarian cancer cells	Induce ROS generation and increase expression of HO-1, a marker of oxidative stress, hence suppression of CDA expression	Yang et al. (2019a)
		Downregulation of CDA causes inhibition of metabolic inactivation of gemcitabine and an overall synergistic effect	
		CI ranges from 0.6–0.9 depending on the concentration ratio which drugs were administered, with an outlier at 1.3 when the ratio of gemcitabine to DHA was 1:1	
	Panc-1and BxPC-3 pancreatic cancer cells	DHA significantly blocks NF-κB activation by gemcitabine, augmenting antitumour effect of gemcitabine	Wang et al. (2010b)
Cisplatin	A549 and A549/DDP NSCLC cells	Increase apoptosis in combination therapy	Zhang et al. (2013b)
		Synergistic effect on inhibition of cell proliferation	
		Combination therapy has lower IC_50_ value compared to monotherapy	
		CI = 0.6706 in A549 and 0.5674 in A549/DDP.	
Cytarabine	HEL92.1.7, MV4-11, U937, ML-2, M07e, MOLM-13, CMK, CMS, mFLT3, MOLM-13-RES, and M07e acute myeloid leukaemia cells	Potentiate cytarabine activity	Drenberg et al. (2016)
		Synergistic effect in MV4-11 and ML-2 cells	
		Better synergistic effect observed when DHA was administered as a pre-treatment, followed by cytarabine	
5-fluorouracil	HCT116, HCT116 TP53^−/−^, SW480, and HT29 colorectal cancer cells	DHA potentiates antitumour activity of 5-FU, combination therapy causes stronger cytotoxic effects and decreases IC_50_ values, even for HCT116 TP53−/− which is resistant to 5-FU.	Yao et al. (2018b)
		Combination therapy reduces number of reproducing HCT116 TP53−/− cells	
		Increase generation of ROS intracellularly, inducing apoptosis	
Carboplatin	A2780 and OVCAR-3 ovarian carcinoma cells	Decrease viability when used in combination–by 69% in A2780 cells, and by 72% in OVCAR-3 cells	Chen et al. (2009b)
		Synergistic increase in apoptosis of OVCAR-3 cells	
		Additive effect of on A2780 cells	
Dictamnine	A549 lung cancer cells	DHA enhances cytotoxicity induced by dictamnine	An et al. (2013)
		DHA enhances apoptosis induced by dictamnine by the caspase-3 dependent pathway	
Apo2L/TRAIL	PANC-1 and BxPC-3 pancreatic cancer cells	Synergistic inhibition of growth	Kong et al. (2012)
		DHA enhances apoptosis induced by Apo2L/TRAIL by ROS pathway	
		Combination index <1 indicating synergistic effect	
Gefitinib	NCI-H1975 NSCLC cells	Potentiates apoptotic effect of gefitinib	Jin et al. (2017)
		Potentiates effect of gefitinib on downregulation of expression of Cdk1 and cyclin B1	
		Enhanced effect of gefitinib on inhibition of cell migration and invasion	
		Enhanced effect of gefitinib on downregulation of p-Akt, p-mTOR and p-STAT3	
		Enhanced effect of gefitinib on upregulation of Bax and downregulation of Bcl-2	
Arsenic Trioxide	A549 lung cancer cells	Synergistic effect on cell viability	Chen et al. (2017a)
		Synergistic effect on DNA damage	
		Synergistic effect on ROS production intracellularly	
		Synergistic effect in inducing apoptosis and cell cycle arrest	
Onconase	A549 NSCLC xenograft	Mice that were treated with combination (onconase 3 mg/kg followed by DHA 10 mg/ml the next day) experienced enhanced suppression of tumour growth and angiogenesis	Shen et al. (2016)
		Mean body weight only slightly changed and no obvious adverse effects observed	
Gemcitabine	A2780 ovarian cancer xenograft	Mice that were treated with combination (DHA 95 mg/kg and gemcitabine10 mg/kg) injected on days 0, 3, 6, and 9 experienced an enhanced effect on inhibition of tumour growth leading to complete elimination of tumour	Yang et al. (2019a)
		No change in body weight	
Carboplatin	A2780 and OVCAR-3 ovarian cancer xenograft	Mice that were treated with the combination (DHA 10 or 25 mg/kg/5 days/week for 3 weeks with carboplatin at a single dose of 120 mg/kg, once on day 0) experienced enhanced inhibition of tumour growth (70%) in both A2780 and OVCAR-3 models, as compared to monotherapy with DHA (41% in the A2780 xenograft and 37% in the OVCAR-3 xenograft) with minimal change in body weight	Chen et al. (2009b)
		Decrease in Bcl-2/Bax ratio and pro-caspase 8	
Cisplatin	A549 and A549/DDP NSCLC xenografts	Mice that were treated with combination of cisplatin (2 mg/kg/3days) and DHA (50, 100, or 200 mg/kg/day) were demonstrated to have greater suppression of VEGF expression and significant decrease in the number of blood vessels compared to monotherapy	Zhang et al. (2013b)
		DHA enhanced chemotherapeutic effect of cisplatin resulting in significant regression compared to monotherapy	
		Increasing doses of DHA also increased the concentration of cisplatin in tumour cells	
Doxorubicin	HeLa cervical cancer heterologous tumour model	Mice that received combination therapy (15 mg/kg DHA and 15 mg/kg doxorubicin) experienced synergistic inhibition of tumour size and more significant reduction in size	Tai et al. (2016)
		No toxicity observed in heart, spleen, liver, and kidneys, and no change in weight	
Apo2L/TRAIL	BxPC-3 pancreatic cancer xenograft	Mice that received combination therapy (DHA 10 mg/kg/day and Apo2L/TRAIL 50 µg/day) experienced a significantly larger reduction in tumour volume compared to those that received DHA or Apo2L/TRAIL monotherapy	Kong et al. (2012)
		DHA potentiates antitumour effect of Apo2L/TRAIL.	
		Combination therapy had higher apoptosis and lower expression of PCNA, a cell proliferation marker, than monotherapy	
**Drugs:artesunate**			
Cisplatin	A549 lung cancer cells	Synergistic effect on antiproliferation induced by cisplatin	Li et al. (2021b)
		CI values < 1, CI values decrease as concentration of drugs increase	
		ART sensitised A549 cancer cells to apoptosis and G2/M cell cycle arrest induced by cisplatin	
		Upregulation of expression of P21, P53, and Bax, and downregulation of expression of Bcl-2 in combination treatment	
		Increase caspase activity in combination therapy	
Bortezomib	MV4-11 acute myeloid leukaemia cells	Synergistic effect on antiproliferation, apoptosis, and autophagy	Hu et al. (2019)
		Upregulation of pro-apoptotic protein Bim and autophagy related protein LC3B in combination therapy	
		Increase activation of caspases	
		Downregulate expression of Bcl-2	
Bromocriptine	GH3 and MMQ rat pituitary adenoma cells	Synergistic effect on cell growth inhibition and inducing cell death Synergistic effect on reduction of cell viability	Wang et al. (2017)
		Inhibit cell proliferation and G1-phase cell cycle arrest	
		Combination therapy induced apoptosis in a caspase-dependently	
Triptolide	PANC-1, CFPAC-1 pancreatic cancer cells	Enhanced inhibitory effects and synergistic effect on cell viability	Liu and Cui, (2013)
		Synergistic effect on activation of caspases and hence apoptosis	
		Synergistic effect on downregulation of heat shock proteins Hsp20 and Hsp27	
Doxorubicin	J16, CEM, Molt-4, Hut78, J-Neo, J-Bcl-2, J-caspase-8^−/−^, Jurkat A3 FADD^−/-^, parental Jurkat A3, and CEM-Dox_R_ leukaemia cells	Synergise to enhance apoptosis	Efferth et al. (2007)
Sorafenib	Caki-1, 786-O, and SN12C-GFP metastatic renal cell carcinoma cells	Synergistic effect on cytotoxicity	Jeong et al. (2015)
		Sorafenib sensitises RCC cells to oxidative stress mediated by ART.	
	SK-hep1 and SM-7721 hepatocellular carcinoma cells	Synergistic effect on apoptosis due to dual inhibitory effects on RAF/MAPK and PI3K/AKT/mTOR pathways	Yao et al. (2020)
		Combination index <1	
Temozolomide	LN229, A172, and U87MG glioblastoma cells	ART enhances cell death induced by temozolomide	Berte et al. (2016)
Allicin	MG-63, U20S, 143-B, SaOS-2 and HOS osteosarcoma cells	Synergistic effect on inhibition of cell viability	Jiang et al. (2013)
		Synergistic effect on induction of apoptosis	
		Upregulation of caspase activation in combination therapy	
Oxaliplatin	MCF7 breast cancer, HCT116 colon cancer and A549 lung cancer cells	ART exerts additive effect to reduce cell number and cell viability	Liu et al. (2011)
Lenalidomide			
		Lenalidomide enhanced effect of ART on A549 and MCF7 cells	
Gemcitabine			
Rituximab	Malignant B cells	Rituximab increases susceptibility of ART-induced apoptosis	Sieber et al. (2009)
Cytarabine	HEL92.1.7, MV4-11, U937, ML-2, M07e, MOLM-13, CMK, CMS, mFLT3, MOLM-13-RES, and M07e acute myeloid leukaemia cells	Synergistic effect when administered both simultaneously and sequentially	Drenberg et al. (2016)
		Combination therapy enhanced antileukemic activity	
Cisplatin	A549 lung cancer xenograft	ART sensitises A549 cells to cisplatin and combination treatment of cisplatin at 3 mg/kg/dose every 3 days and ART at 200 mg/kg/dose daily orally for 3 weeks. led to a more significant inhibition of tumour growth than monotherapy	Li et al. (2021b)
		No difference in body weight in combination therapy	
Allicin	MG-63 human osteosarcoma xenograft	Mice that received the combination therapy of ART 50 mg/kg OD and allicin 5 mg/kg OD had significantly suppressed tumour growth compared to monotherapy	Jiang et al. (2013)
Cytarabine	MV4-11-luc, ML-2, and MOLM-13 acute myeloid leukaemia xenografts	Mice that received the combination therapy of ART 120 mg/kg/day for 5 days and cytarabine 6.25 mg/kg/day for 5 days experienced a decrease leukemic infiltration though there was no prolonging of overall survival rate	Drenberg et al. (2016)
Sorafenib	SK-7721 HCC xenograft	Combined treatment of sorafenib 2.5 mg/kg and ART 100 mg/kg reduced tumour growth to a larger extend than monotherapy	Jing et al. (2019)
	786-O metastatic RCC xenograft	ART potentiates antitumour effects of sorafenib	Jeong et al. (2015)
Temozolomide	U87MG glioblastoma xenograft	Repeated concomitant treatment extended mean survival period	Berte et al. (2016)
		Combination treatment of temzolomide 5 mg/kg 5 times a week for 6 weeks and ART 100 mg/kg for 9 weeks inhibited tumour growth more effectively than monotherapy	
Triptolide	PANC-1 and CFPAC-1 pancreatic cancer xenograft	Mice that received combination therapy (triptolide 50 μg/kg and ART 50 mg/kg, OR triptolide 50 μg/kg and ART 100 mg/kg, OR triptolide 100 μg/kg and ART 50 mg/kg, OR triptolide 100 μg/kg and ART 100 mg/kg experienced synergistic effect on inhibition of tumour growth which caused greater decrease in tumour size than monotherapy	Liu and Cui, (2013)
		No significant change in body weight in combination treatment	

Animal xenograft models showed that the combination of artemisinins with onconase (Shen et al., 2016), gemcitabine (Yang et al., 2019a), carboplatin (Chen T. et al., 2009), cisplatin (Zhang YJ. et al., 2013; Li W. et al., 2021), doxorubicin (Tai et al., 2016), Apo2L/TRAIL (Kong et al., 2012), allicin (Jiang et al., 2013), cytarabine (Drenberg et al., 2016), sorafenib (Jeong et al., 2015; Jing et al., 2019), triptolide [17], and temozolomide (Berte et al., 2016) can exert a synergistic effect on leukemia (Drenberg et al., 2016), renal cell carcinoma (Jeong et al., 2015), glioblastoma (Berte et al., 2016), lung (Zhang YJ. et al., 2013; Shen et al., 2016; Li W. et al., 2021), ovarian (Chen T. et al., 2009; Yang et al., 2019a), cervical (Tai et al., 2016), pancreatic (Kong et al., 2012; Liu and Cui, 2013), and liver (Jing et al., 2019) cancer. Many studies reported the synergistic effect of ART with conventional chemotherapy on the inhibition of tumor growth without a significant decrease in body weight (Kong et al., 2012; Liu and Cui, 2013; Shen et al., 2016; Tai et al., 2016; Yang et al., 2019a; Li W. et al., 2021), suggesting improved efficacy without an overt increase in toxicity. The complete elimination of an ovarian cancer tumor was observed in a study that used DHA and gemcitabine combination therapy.

In summary, combination therapy is a promising strategy to advance the repurposing of artemisinins as anticancer therapeutics. Since more combination therapy studies have been conducted for DHA than for ART, the use of DHA in human clinical trials should also be explored in future research. Clinical trials exploring ART or DHA as an adjuvant to the conventional chemotherapy should also be conducted.

### Nanoformulation

To overcome the limitations that result from poor pharmacokinetic properties of artemisinins, novel delivery methods that could improve the absorption and elimination profile of artemisinins should be explored. Several *in vitro* and *in vivo* studies have been conducted to investigate the use of nanoparticles, nanocarriers, and liposomes as carriers for ARS, ART, and DHA to improve their delivery to the cancer cells. These new formulations improved solubility, exposure, and stability, increased cellular uptake, and enhanced permeability and retention in breast, colorectal, liver, lung, and cervical cancer cells (Chen J. et al., 2014; Chen et al., 2015; Tran et al., 2015; Leto et al., 2016; Liu et al., 2016; Tran et al., 2016; Tran et al., 2017; Wang et al., 2018; Wang et al., 2019; Phung et al., 2020). Both *in vitro* and *in vivo* studies revealed promising results with low IC_50_ values (Zhang et al., 2015; Leto et al., 2016) and high rates of tumor inhibition (Jin et al., 2013; Chen et al., 2015; Wang et al., 2016b; Liu et al., 2016; Dong et al., 2019; Wang et al., 2019; Li et al., 2020).

In a study conducted on BT474 (HER2+) breast tumor cells made using liposomal nanoparticles for drug delivery, IC_50_ values ranged between 0.07–0.39 µM (Zhang YJ. et al., 2013), indicating high potency. In another study, IC_50_ values decreased from 127 ± 8.5 µM when free ARS was administered to 69 ± 23 µM when liposomes were administered (Leto et al., 2016), demonstrating the ability of liposomes to increase the efficacy of ARS. Many formulations used pH-dependent drug release in the slightly acidic environment of tumor cells (Wang et al., 2016a; Wang et al., 2016b; Dong et al., 2019; Wan et al., 2019; Wang et al., 2019) for targeted drug delivery and increased accumulation of the drug in the tumor cells while simultaneously reducing unintended off-target interactions. This might have contributed to the greater cytotoxicity observed with the use of novel nanoformulations than with the use of free drug (Chen J. et al., 2014; Chen et al., 2015; Tran et al., 2015; Wang et al., 2016b; Tran et al., 2017; Dong et al., 2019).

After nanoformulation administration, the same efficacy was demonstrated in *in vivo* studies, whereas an increase in antitumor effect was observed in tumor-bearing mice models (Jin et al., 2013; Chen et al., 2015; Zhang et al., 2015; Wang et al., 2016a; Wang et al., 2016b; Liu et al., 2016; Wang et al., 2018; Dong et al., 2019; Wang et al., 2019; Li et al., 2020; Phung et al., 2020). Antitumor effect was measured by using the tumor volume and tumor growth inhibition rate. In a study that used nanoconjugates, breast tumor volume was 989 ± 164 mm^3^ after treatment with nanoconjugate formulation compared to 1,417 ± 148 mm^3^ after treatment with the free drug (Li et al., 2020). Another study conducted on Lewis lung carcinoma tumor bearing mice model reported a tumor growth inhibition rate of 84.6% after treatment with polyethylene DHA nanoparticles compared to 29.9% after treatment with free DHA. Survival rate was also markedly higher (83.3%) than that of free DHA (16.7%) (Liu et al., 2016).

In the future research, combination therapy and nanotechnology should be further explored. The combinations of DHA with oxaliplatin (Duan et al., 2019), DHA with sorafenib (Wang et al., 2019), DHA with docetaxel (Li et al., 2020), and DHA with paclitaxel (Phung et al., 2020) along with the use of nanoparticles have been studied, and *in vitro* and *in vivo* data are promising, implying their viability for human trials.

## Concluding Remarks

Despite challenges, repurposing artemisinins for cancer treatmentis possible. Artemisinin and its derivatives have anticancer effects against multiple cancer types. because they act through various pathways, although their potency varies across cancer types. Their efficacy has also been demonstrated in *in vivo* studies with evidence of inhibition of tumor growth in tumor bearing mice models. A few human trials have also shown promising results that artemisinins, in particular ART, are safe for use, although their efficacy is still relatively limited. The limitations due to their pharmacokinetic properties such as low tissue distribution, short half-life, and unpredictable toxicity at high doses hinder their clinical translation. However, there are viable options such as the use of combination therapy and nanoformulations that can overcome the pharmacokinetic barriers of artemisinins. At high doses of artemisinins are used in cancer treatment, toxicity prediction models should be used to ensure that severe toxicity is controlled (Li S. et al., 2021). Although artemisinins have great potential as anticancer agents, additional extensive human trials are required before the drug can be established as an anticancer agent.

## Data Availability

The raw data supporting the conclusions of this article will be made available by the authors, without undue reservation.
